# Adaptation to Progressive Additive Lenses: Potential Factors to Consider

**DOI:** 10.1038/s41598-017-02851-5

**Published:** 2017-05-31

**Authors:** Tara L. Alvarez, Eun H. Kim, Bérangère Granger-Donetti

**Affiliations:** 1 Department of Biomedical Engineering, New Jersey Institute of Technology, Newark, NJ 07102 USA; 2 Department of Vision Sciences, Essilor Research & Development, Paris, France

## Abstract

People develop presbyopia as part of the normal aging process. Most presbyopes adapt to progressive additive lens (PALs), while others do not. This investigation sought to determine whether the ability to modify disparity vergence or phoria was correlated to PALs adaptation. In experiment 1, a double-step paradigm quantified the ability to modify convergence responses in sixteen presbyopes. In experiment 2, thirty-one incipient presbyopes participated in a 5-minute sustained fixation task to evoke phoria adaptation where the magnitude and rate of phoria adaptation were measured. Then, the experiment was repeated after wearing PALs for one month. Linear regression analyses were conducted between the following parameters: near point of convergence, positive fusional vergence at near, vergence facility, net change in the magnitude of phoria adaptation, and the rate of phoria adaptation. The ability to change convergence average peak velocity was significantly greater (*p* < 0.03) in presbyopic PALs adapters compared to presbyopic PALs non-adapters. The rate of phoria adaptation and vergence facility were significantly greater (*p* < 0.03) in incipient presbyopic PALs adapters compared to incipient presbyopic PALs non-adapters. Vergence facility and the rate of phoria adaptation may have potential clinical utility in differentiating which patients may adapt to PALs and which ones will have more difficulty.

## Introduction

Presbyopia is a part of the natural aging process that all humans experience and is the gradual loss of accommodative amplitude as a function of increasing age^1^. Age-related decreases in the ciliary muscle length (extralenticular process)^2^, hardening of the lens^3^, increase in viscosity^4^, stiffness of the lens^5^, and geometric changes such as the increase in lens thickness and convexity^6, 7^ are all prominent contributors to the loss of accommodative amplitude. Other factors that influence the onset of presbyopia include focusing ability, depth of focus^8^, refractive error^9^, pupil size^10^, and environmental factors such as humidity or levels of ambient light^11^. Loss of accommodation is gradual, with first visual symptoms occurring between 42 and 44 years of age^12^. The vast majority of the human population experiences visual symptoms by the age of 51 years^13^. Within the aging population, it is estimated that 1.4 billion people will be affected by presbyopia by the year 2020 with this number increasing to 1.8 billion by 2050^14^.

Presbyopia is often overlooked as a health condition since corrective modalities such as presbyopic spectacle lens corrections, contact lenses, or intraocular lenses are readily available and provide adequate vision over a range of distances. Corrective presbyopic spectacle lenses include several commonly prescribed implements such as reading glasses, bifocal lenses, and progressive lenses. Conventional bifocal lenses offer two zones of fixed-focus vision (distance and near) separated by a visible ‘line’. The discontinuity between the two lenses produces an abrupt change in the line of sight and thus stimulates a prismatic effect^15^. Alternatively, progressive addition lenses (PALs) are multifocal lenses that provide the desired addition power by continuously increasing the power between the distance and near zones on the surface of the lens^16, 17^. PALs promote alternating vision through different lenses as opposed to the simultaneous vision used by multifocal intraocular or contact lenses.

Because bifocals are often associated with old age, many presbyopes prefer PALs over bifocals. However, some presbyopes have difficulty when wearing PALs and may experience moderate to severe visual symptoms such as blurred vision, headaches, perceived movement of the peripheral visual field or “swim”, balance issues, and nausea^18^. Eventually, these individuals discard PALs and use other corrective modalities such as reading glasses. It is not completely understood in the literature as to why some presbyopic individuals have such difficulty adapting to PALs while others adapt to PALs with ease. Thus, understanding potential underlying mechanisms of PALs acceptability has significant clinical impact for the growing presbyopic population who will require accommodative correction.

Accommodation is described by Maddox and later defined by Heath as using the following four components as its primary stimuli: tonic accommodation, proximal accommodation, blur-driven accommodation, and convergence accommodation^19^. Chromatic aberration has also been shown to drive accommodation for both moving and stationary targets^20^. Maddox similarly described vergence to have tonic, proximal, blur and disparity inputs^21^. Vergence is the inward (convergence) or outward (divergence) rotation of the eyes to project an object of interest onto the fovea, the highest acuity portion of the retina. The two main inputs to the vergence system are accommodation (via blur) and disparity. In most models of the oculomotor system, accommodation is not a stand-alone component but rather interacts with vergence via neural crosslinks between the two systems^22, 23^. These interactions are readily demonstrated in the form of accommodative convergence and convergence accommodation, which are measured as the AC/A ratio and CA/C ratio, respectively^22–25^. Thus, as accommodative convergence decreases with the onset of presbyopia^26^ the role of disparity-vergence gains importance in the maintenance of single binocular vision. Single binocular vision is described as a state of simultaneous vision coordinated through the use of both eyes such that separate images are observed as a single image via the process of fusion^27–30^. Previous studies have suggested that phoria adaptation, or the ability to sustain consistent eye alignment in the presence of a binocular stimulus, may also be a primary factor in maintaining stable and comfortable binocular vision^28–30^. These studies have shown a clear relationship between disparity convergence and phoria, where the change in the magnitude of phoria reduces the load on the disparity convergence system. However, prior studies have yet to examine whether disparity-vergence and phoria were correlated to the successful adaptation to PALs among the presbyopic population.

The purpose of this study is to investigate whether the ability to modify the vergence and phoria systems is significantly different between presbyopes who could not adapt to PALs and presbyopes who have successfully adapted to PALs and wear them regularly. Specifically, this study will examine whether a greater change in convergence peak velocity during a short-term modification experiment and faster rate of phoria adaptation may differentiate adapters from non-adapters of PALs. Adaptation to PALs will be defined in this study as the subject’s self-report of whether s/he will continue to wear PALs daily. Moreover, this knowledge may provide insight regarding the successful adaptation to PALs, thus allowing clinicians to better prescribe treatments suited to the individual to reduce visual symptoms associated with presbyopia. Using the knowledge acquired from the proposed experiments described below, this study seeks to determine which parameter(s) from the vergence and phoria systems best correlated with PALs acceptability.

## Methods

### Overview

Subjects: A total of 50 subjects were recruited for this study where 47 completed data collection. All experimental methods were carried out in accordance with the relevant guidelines and were approved by the New Jersey Institute of Technology Institution Review Board. All subjects signed a written informed consent form approved by the New Jersey Institute of Technology Institution Review Board in accordance with the Declaration of Helsinki. All subjects were free of neurological dysfunction or injury, as well as ocular, oculomotor, or binocular abnormalities. All subjects wore standard traditional PALs as opposed to digital or free-form PALs.

Common Visual Parameter Measurements: All subjects saw the same licensed Optometrist who has over 30 years of experience and ensured that correction to normal vision was achieved. Objective measures of refraction were obtained using static retinoscopy. All subjects had corrected-to-normal vision during the entire experiment. Binocular function was assessed using a Randot Stereopsis Test (Bernell Corp., South Bend, IN, USA). The test consisted of eight grades that ranged from 20 to 400 seconds of arc. Normal was defined as better than 70 seconds of arc. Near point of convergence (NPC) was measured by having the operator slowly bring a high acuity object (the letter ‘E’) towards the subject along his/her midline. When the subject reported diplopia and was no longer able to fuse the targets then the distance from the bridge of the nose to the target was measured as the NPC. Near fusional vergence was measured with the Bernell horizontal prism bar (Bernell Corp., South Bend, IN, USA) to assess both positive and negative fusional range. The bar contained 15 prims: 1Δ, 2Δ through 20Δ in 2Δ increments, and 20Δ to 45Δ in 5Δ increments. The subject held a high acuity object (the letter ‘E’) 40 cm away along his/her midline. The operator held the prism bar over the subject’s right eye. The operator instructed the subject to keep the images single and report when he/she was no longer able to perceive the letter ‘E’ as single. A new prism was introduced about every 2 seconds. Positive and negative fusional range was assessed as the largest base-out and base-in prisms, respectively that a subject could maintain single vision. These methods have been used in our prior studies from this laboratory^31–42^.

Instrumentation: Convergence eye movements were recorded using a video-based eye movement monitoring system (λ = 950 nm) manufactured by ISCAN (Massachusetts, USA). Eye movement responses were sampled at 240 Hz using a 12-bit digital acquisition hardware card (National Instruments 6024 E series, Austin, TX, USA). The left-eye and the right-eye responses were recorded, calibrated, and saved separately for offline data analysis. The entire system was controlled by a custom LabVIEW program (National Instrument, Austin, TX, USA)^43, 44^. Stimuli were presented on a haploscope and were 2 cm in height and 2 mm in width. The ISCAN eye movement tracking system has a resolution of 0.1° with a linear range of 3% for horizontal movements from ±25°. Our prior studies have analyzed linearity and report a Pearson correlation coefficient of r > 0.9 for our calibrations^45^. Our prior studies also show a high interclass correlation coefficient of >0.7 when assessing eye movements within and between experimental trials^46–48^.

There were two types of experiments within this study: the short-term vergence modification experiment 1 and the phoria adaptation experiment 2 which will be described in detail below.

### Experiment 1 Short-term Vergence Modification Experiment

#### Objective Description

The purpose of the short-term vergence modification experiment is to determine whether the ability to modify convergence responses differ between adapters and non-adapters to PALs.

#### Subjects

**Figure 1:**
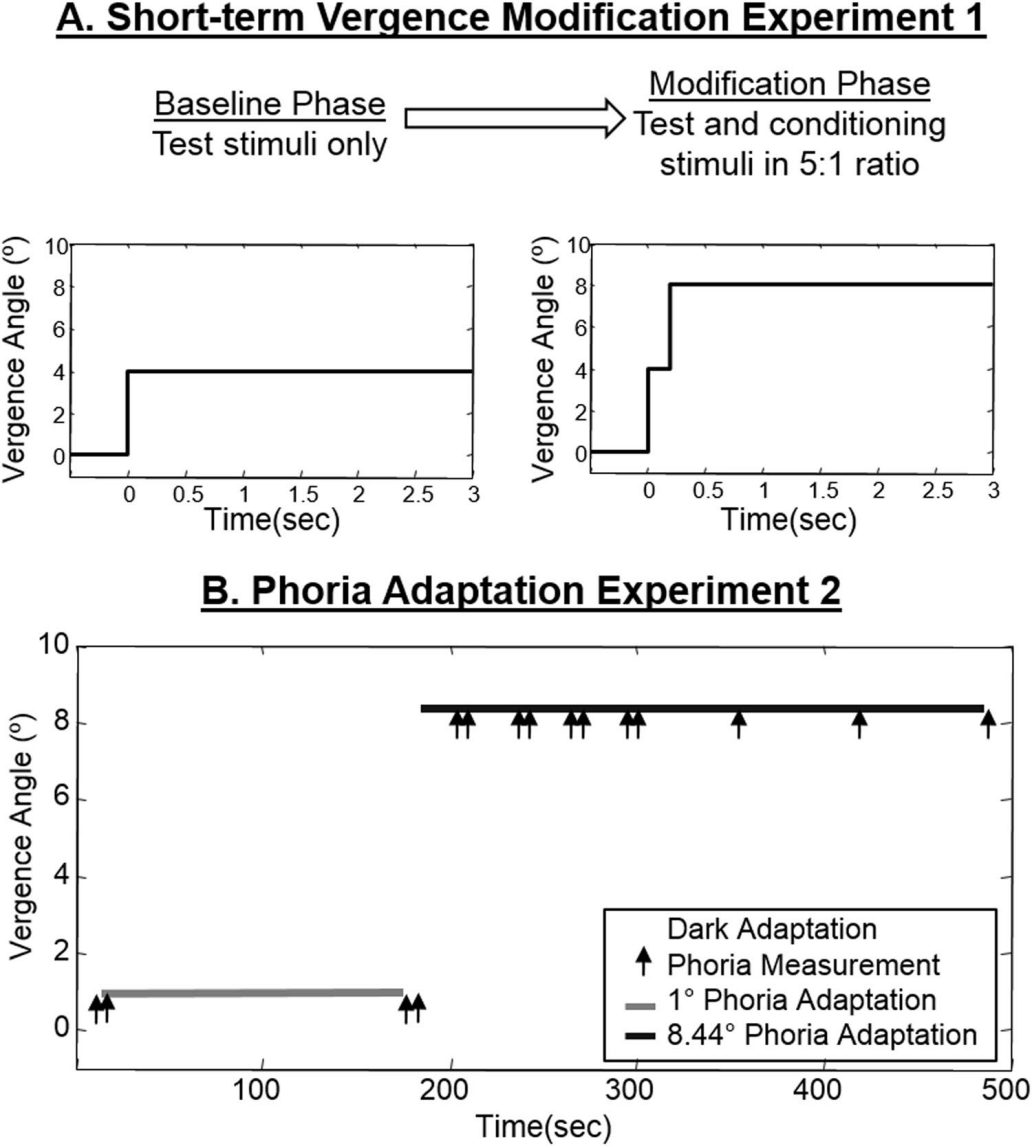
Experimental protocol of (**A**) the short-term vergence modification for Experiment 1 and (**B**) the phoria adaptation for Experiment 2.

Sixteen presbyopic subjects participated in the short-term vergence modification experiment. Eight subjects reported wearing progressive lenses regularly; these subjects were referred to as PAS (presbyopic adapting subjects). Eight subjects reported trying progressive lenses but could not adapt to wearing PALs and subsequently terminated use; period of use varied in length among these subjects, ranging from 1 to 3 months. These subjects were referred to as PNAS (presbyopic non-adapter subjects). It is potentially possible that if subjects who reported they could not adapt to PALs were to wear PALs longer then s/he may have eventually adapted to them. However, it is beyond the scope of this study to determine whether subjects who self-reported that they could not adapt to PALs after 1–3 months would adapt to PALs if they were given more time to wear them. Presbyopes who could not adapt to PALS typically used multiple glasses to perform near versus far work wearing prescription lenses prescribed by a licensed optometrist. **Experimental Protocol:** The experiment consisted of two phases: the baseline and modification phase. Two types of convergence stimuli termed as test and conditioning stimuli shown in Fig. 1 plot A were presented to the subjects. During the baseline phase, the subj ects viewed only the test stimuli (4° symmetrical convergence step stimuli). Subsequently, in the modification phase, the subjects viewed the conditioning stimuli (4° symmetrical double step with a 200 msec delay between steps for a total change in disparity of 8°) and the test stimuli in a five to one ratio. This experiment was designed to observe how the conditioning stimuli (double step) influenced the test (single step) responses. The 200 msec delay was chosen because it is less than the typical latency of vergence and has been used in prior short-term modification vergence experiments^28, 39, 49–54^.

#### Data analysis

A custom MATLAB program (Waltham, MA, USA) was used for data analysis. The left and right-eye data were converted into degrees using calibration data. Monocular stimuli were presented into each visual field to calibrate eye movement responses into degree of angular rotation. Convergence was calculated by measuring the difference between the right-eye and the left-eye movements. Responses with saccades in the transient portion of the vergence responses were omitted from further data analyses since saccades have been shown to increase convergence velocity^43, 55–57^. Blinks were rare within the data set since the subjects initiated each trial. Responses with blinks within the transient portion of the response were omitted from data analysis.

Convergence velocities were measured from convergence responses using a two-point central difference algorithm^58^. Peak velocity was identified as the maximum point within the convergence velocity trace as described in our prior research^59^. The change in convergence peak velocity was computed as the average of the 4° convergence peak velocities during the modification phase minus the average of the 4° convergence peak velocities during the baseline phase. The double-step responses contained two peak velocities, which were defined as the first high-velocity component and the second high-velocity component, respectively. The high-velocity ratio was calculated as the ratio of the first high-velocity component divided by the second high-velocity component.

A repeated measures ANOVA was calculated using NCSS2004 (Kaysville, UT, USA) to determine whether 4° convergence steps were significantly different between the baseline and modification phases. A two tailed student unpaired *t*-test was used to determine during the short-term modification experiment whether significant differences would be observed between the PNAS and PAS groups for the following parameters: 1) 4° convergence peak velocity from baseline and modification phases, 2) change in convergence peak velocity, 3) double-step peak velocity, and 4) the high-velocity ratio.

### Experiment 2: Phoria Adaptation Experiment

#### Objective Description

The purpose of the Phoria Adaptation experiment is to determine whether the ability to adapt the phoria level differed between adapters and non-adapters to PALs.

#### Subjects

A total of 34 incipient presbyopes were fitted for progressive lenses for the first time. After approximately one month of wearing progressive lenses (31.7 ± 5.7 days), subjects self-reported whether they had adapted to wearing progressive lenses or not. Adapters were those subjects who were going to continue wearing PALs daily compared to non-adapters who were going to try another optical correction which was typically multiple pairs of spectacles. The ones who adapted to PALs were called incipient presbyopic adapting subjects (IPAS) and those who tried but could not adapt to PALS were called incipient presbyopic non-adapting subjects (IPNAS). Three of the subjects did not return for the follow-up session and these data were removed from all analysis. Seven subjects reported moderate to severe visual complaints after wearing PALs for one month, stating they would use other corrective modalities such as reading glasses or bifocals. These subjects were classified as IPNAS. Twenty-four subjects reported minimal to mild visual complaints when wearing progressive lenses for one month, stating they would wear PALs daily. These subjects were classified as IPAS.

#### Experimental Protocol

The six static clinical measurements described above were obtained at baseline prior to use of PALs. In addition, vergence facility was measured as the number of fusional cycles per minute (cpm) that a person could view a near (40 cm high acuity target along midline) stimulus as single and clear when alternating between 3 base-in (BI) and 12 base-out (BO) prisms. One fusional cycle was defined as fusion of a near target for both the 3 BI and 12 BO prisms^60, 61^. Symptoms were assessed using the convergence insufficiency symptom survey (CISS)^47^. A haploscope set-up was used to control the visual stimuli to both eyes independently. The stimulus was a green vertical line 2 cm in height and 2 mm in width presented on a black background. The visual stimuli were projected to two partially reflecting mirrors from the computer screens into the subject’s line of sight. Prior to the experimental session, the stimuli from the computer screens were carefully adjusted with mirrors to calibrate the visual stimuli with the targets located at measured distances from the subject’s midline. Since our subjects were slightly converged during our experiment, an average inter-pupillary distance of 6 cm was assumed.

Near dissociated phoria (simply called phoria throughout this paper) was measured in a dark environment where the subjects monocularly viewed with the left eye a vertical line positioned 40 cm away (4.22° inward ocular rotation for the left eye) along the subject’s midline. Simultaneously, the right eye viewed a dark field. The right eye movement position was recorded for 15 seconds. A four-point right-eye monocular calibration covering a range of 7∆ esophoria to 16∆ exophoria was used to convert the right eye data to prism diopters. All of our subjects’ phoria level was well within this range. The phoria level was calculated by averaging the last three seconds of the right eye response based upon prior published data from where a steady state is reached within 12 seconds. Examples of phoria responses have been demonstrated in this laboratory’s previous studies^35, 47, 62–64^.

Before the experiment, subjects were dark adapted for 10 min to uncouple the vergence and accommodation systems^65, 66^. Dark adaptation (10 minutes in duration) also reduced visual adaptation stimulated by near or far visual work that may have been performed prior to the experiment. After dark adaptation, two phoria measurements were recorded. Then, the subject fixated on a far target (a 1° binocular target along midline) for 3 minutes. As shown in Fig. 1 plot B, subjects performed sustained fixation to an 8.44° binocular target located 40 cm away on midline for a cumulative total of 5 minutes. For Fig. 1 plot B, a double arrow marker indicates that two phoria measurements were obtained to assess repeatability of the phoria measurements. These repeated phoria measurements were done after 3 minutes of sustained fixation to a 1° target and four periods of sustained fixation to an 8.44° target. For each pair of phoria measurements, significant differences between measurements were not observed (*p* > 0.1). Therefore, the two phoria measurements were averaged after every 30 sec of sustained fixation to an 8.44° binocular target (cumulative fixation time was 2 minutes). The next three phoria measurements were each recorded after 1 minute of sustained fixation to an 8.44° target with a cumulative fixation time of 5 minutes.

#### Data Analysis

A total of seven phoria measurements during the 5-minute sustained fixation to an 8.44° stimulus were fit with an exponential function because the data followed an exponential curve trajectory based upon visual inspection as opposed to a linear trajectory. The time constant is classically used in engineering and physics where it is defined as the time needed for the response to reach 63% of the steady-state was computed^67^. The rate of phoria adaptation (△/min) was calculated as the net change in the magnitude of phoria divided by the time constant. To study the group-level effects, each subject’s seven phoria measurements were subtracted by the subject’s initial phoria measurement. Hence, the initial phoria measurement was adjusted to zero to conduct a group-level analysis.

A repeated measures ANOVA was calculated to determine whether the rate and the change in magnitude of phoria during 5 minutes of phoria adaptation to an 8.44° binocular target 40 cm from midline were significantly different when comparing baseline measurements to those collected after one month of wearing PALs for incipient presbyopes. These data had a normal distribution and each observation was independent. A student’s two tailed unpaired *t*-test was used to determine whether the rate and change in magnitude of phoria were also significantly different between the IPAS and IPNAS groups. The data used for the two tailed *t*-tests had a normal distribution and each observation was independent. Linear regression analyses included: age, stereopsis, near point of convergence, recovery point of convergence, base-in and base-out vergence ranges, vergence facility, and CISS scores, as well as the rate and magnitude of phoria adaptation from sessions 1 (before PALs) and 2 (after one month of wearing PALs). The Pearson’s correlation coefficient was calculated using MATLAB. All figures were generated using MATLAB and Microsoft Excel software.

Conventionally, to study the utility of a method to assess the success or failure of a therapeutic intervention is the assessment of that intervention’s sensitivity and specificity. For this application, a true positive is a non-adapter to PALs correctly identified as a non-adapter, and a true negative is an adapter to PALs correctly identified as an adapter. A false positive is an adapter to PALs incorrectly identified as a non-adapter to PALs and a false negative is a non-adapter to PALs incorrectly identified as an adapter to PALs. Sensitivity is defined as the number of true positive divided by the summation of true positives and false negatives. Specificity is the number of true negatives divided by the summation of true negatives and false positives. Sensitivity and specificity will be computed using a total of 100 thresholds ranging from the group minimum to group maximum for each parameter investigated. Therefore, a total of 100 sensitivity and 100 specificity values will be calculated. The null hypothesis is that non-adapters will have measurements lower than the 100 thresholds used, while the alternative hypothesis is that adapters will have measurements equal to or greater than the threshold for each parameter studied. Accuracy is defined as the summation of true positives and true negatives divided by the summation of all observations reported as a percentage. The optimal threshold of each of the six parameters is determined based upon the highest combination of sensitivity and specificity assessed using receiver operator characteristic (ROC) curves.

## Results

### Subject Attributes

The clinical measurements and group demographics for Experiments 1 and 2 are shown in Tables 1 and 2, respectively. The first experiment studied presbyopes who had a history of wearing PALs. The second experiment was a longitudinal experiment that investigated incipient presbyopes who were who were fitted for PALs for the first time. For Experiments 1 and 2 there were no significant differences (p > 0.06) observed with a two tailed t-test for stereopsis, near point of convergence, vergence range, left or right sphere, near addition lens, or interpupillary distance when comparing the non-adapter or the adapter of PALs groups.

**Table 1:** Mean and Standard Deviation of Non-Adapters and Adapters to PALs Group Attributes for Experiment 1 Short-term Vergence Modification Experiment studying presbyopes with a history of wearing PALs.

	Experiment 1	
Clinical Parameters	Non-Adapters	Adapters
Age (years)	55.75 ± 8.65	56.38 ± 9.78
Gender (F = Female, M = Male)	2 F and 6 M	1 F and 7 M
Stereopsis (sec arc)	39.3 ± 18.6	36.6 ± 14.8
Near Point of Convergence (cm)	7.4 ± 1.6	7.7 ± 2.7
Base-In Vergence Range (△)	14.43 ± 6.43	12.25 ± 3.11
Base-Out Vergence Range (△)	35.00 ± 6.45	32.5 ± 7.56
Mean Right Eye Sphere O.D. (D = Diopters)	−0.21 ± 2.5	0.53 ± 2.7
Mean Left Eye Sphere O.S. (D = Diopters)	0.07 ± 3.0	0.60 ± 2.8
Near Addition (D = Diopters)	1.50 ± 0.5	1.90 ± 0.9
Interpupillary distance (mm)	65.5 ± 6.0	66.4 ± 5.7

**Table 2:** Mean and Standard Deviation of Non-Adapters and Adapters to PALs Group Attributes for Experiment 2 Phoria Adaptation Experiment studying incipient presbyopes who were fitted for PALs for the first time.

	Experiment 2	
Clinical Parameters	Non-Adapters	Adapters
Age (years)	48.57 ± 2.99	46.50 ± 3.28
Gender (F = Female, M = Male)	6 F and 1 M	14 F and 10 M
Stereopsis (sec arc)	36.4 ± 9.5	36.0 ± 18.8
Near Point of Convergence (cm)	6.0 ± 2.1	6.7 ± 2.6
Base-In Vergence Range (△)	10.00 ± 3.46	11.00 ± 3.24
Base-Out Vergence Range (△)	29.57 ± 15.09	34.54 ± 12.42
Mean Right Eye Sphere O.D. (D = Diopters)	−2.92 ± 3.1	−1.03 ± 2.0
Mean Left Eye Sphere O.S. (D = Diopters)	−2.04 ± 2.4	−0.71 ± 1.7
Near Addition (D = Diopters)	1.71 ± 0.4	1.68 ± 0.5
Interpupillary distance (mm)	62.8 ± 3.6	64.4 ± 4.3

### Differences in Vergence Peak Velocities in PNAS and PAS Groups (Experiment 1)

**Figure 2:**
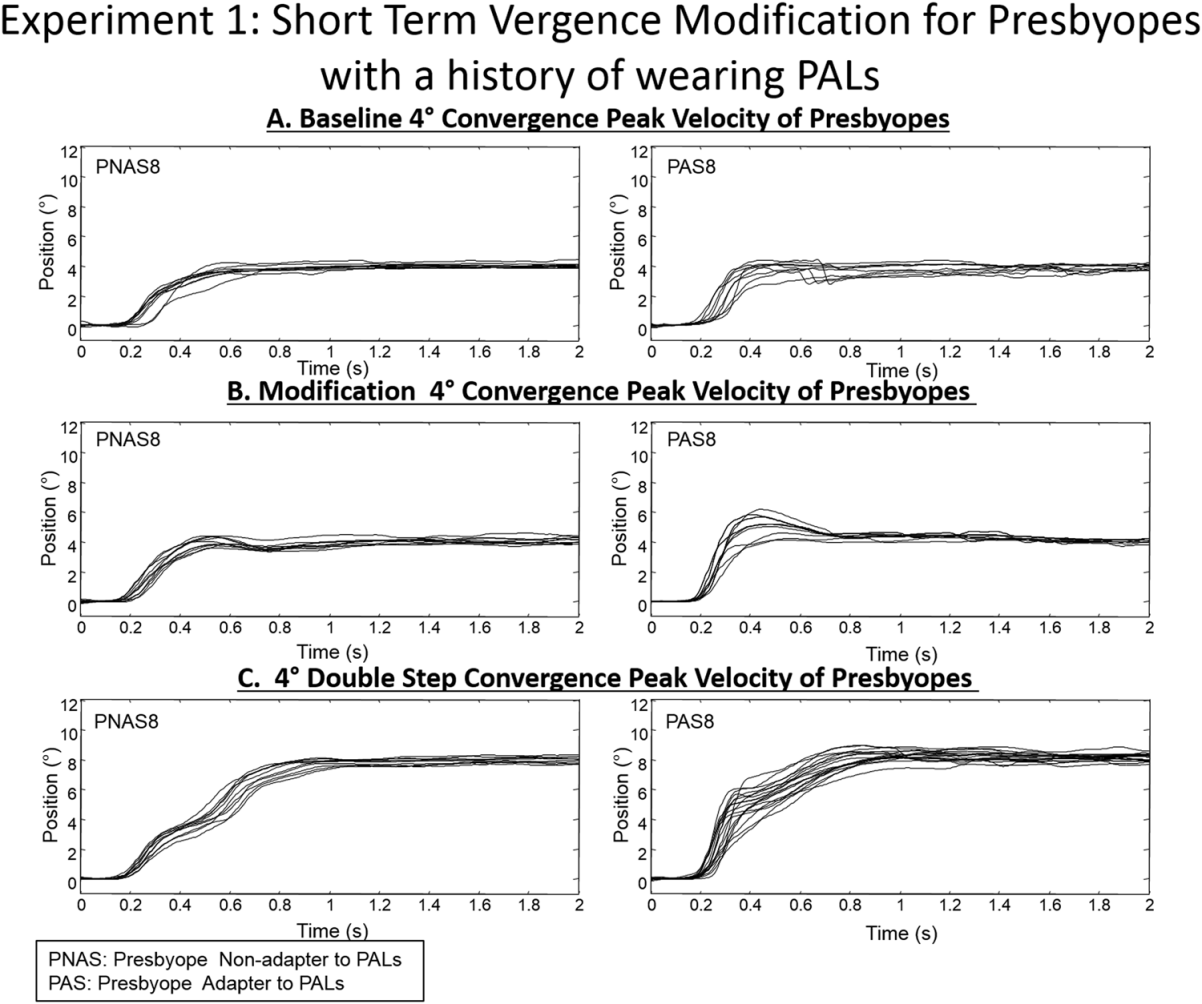
Ensemble plots for 4° single and double convergence responses from a subject from the PNAS (left column) and PAS (right column) groups for Experiment 1.

**Figure 3:**
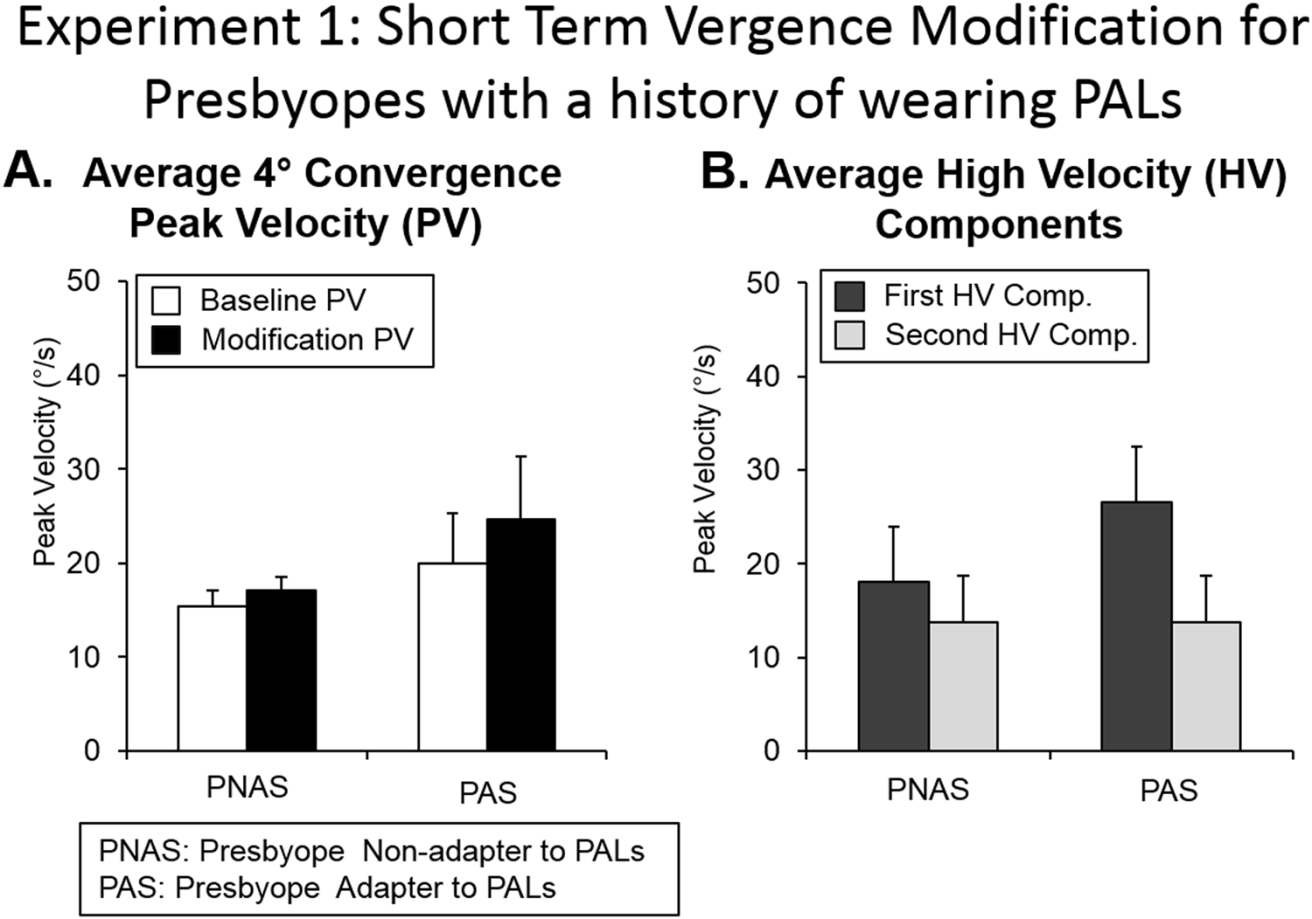
Summary of the average peak velocity (°/s) with one standard deviation of (**A**) 4° convergence steps during baseline and modification phases (**B**) first and second high-velocity components during the modification phase. Individual subject data from eight subjects who did not adapt to PALs (PNAS) and eight subjects who did adapt to PALs (PAS) were averaged.

In Fig. 2, single-subject ensemble plots are shown for the following eye movement responses: baseline 4° symmetrical convergence steps, modification 4° symmetrical convergence steps, and 4° symmetrical convergence double steps (PNAS, left column; PAS, right column). Group averages of 4° convergence peak velocities from baseline and modification phases are summarized in Fig. 3A. An unpaired *t-*test revealed statistical differences in 4° convergence peak velocities between groups during both baseline (T = 2.28, *p* = 0.04*)* and modification (T = 3.14, *p* < 0.01) phases. PNAS subjects exhibited slower 4° convergence responses during baseline and modification phases compared to PAS subjects. An unpaired *t*-test revealed statistically significant differences for the first high-velocity component (T = 2.86, *p* = 0.013) but showed no significant differences for the second high-velocity component (T = 0.02, *p* = 0.98) (Fig. 3B). For the first high-velocity component, PNAS subjects exhibited slower high-velocity components when compared to the PAS subjects.

**Figure 4:**
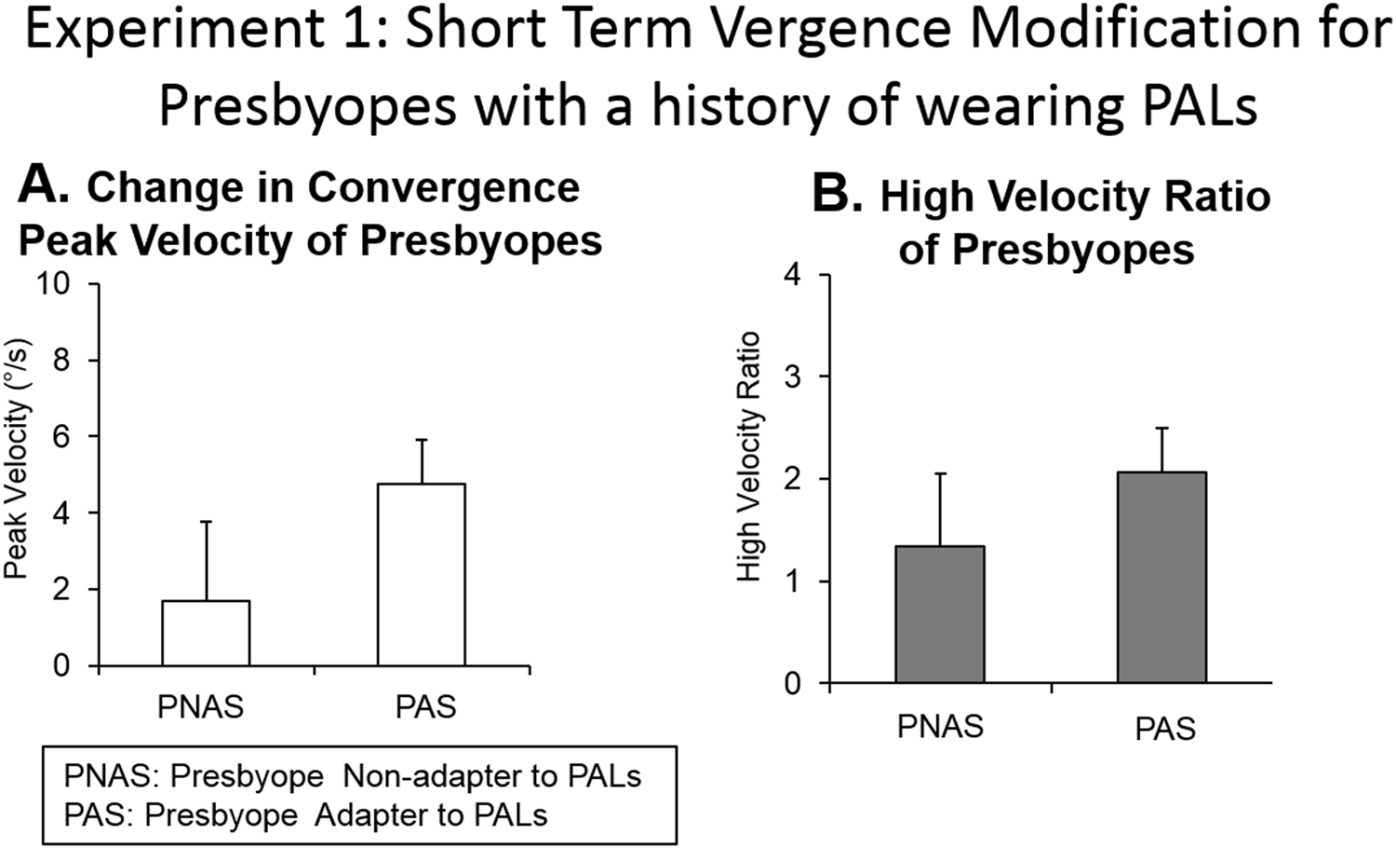
(**A**) Change in convergence peak velocity and (**B**) High-velocity component ratio for the eight individual subjects who did not adapt to PALs (PNAS) and the eight subjects who adapted to PALs (PAS). Group level summary plots are shown as the average with one standard deviation.

Figure 4A demonstrates a significantly greater change in 4° convergence peak velocity among PAS subjects as compared to PNAS subjects (T = 3.66, *p* = 0.002). The high-velocity ratio was quantified as the first high-velocity component divided by the second high-velocity component. The high-velocity ratios between PNAS and PAS were also significantly different (T = 2.43, *p* = 0.030) as shown in Fig. 4B, where the ratio was greater in PAS compared to PNAS.

### Differences in Phoria Adaptation in IPNAS and IPAS Groups (Experiment 2)

**Figure 5:**
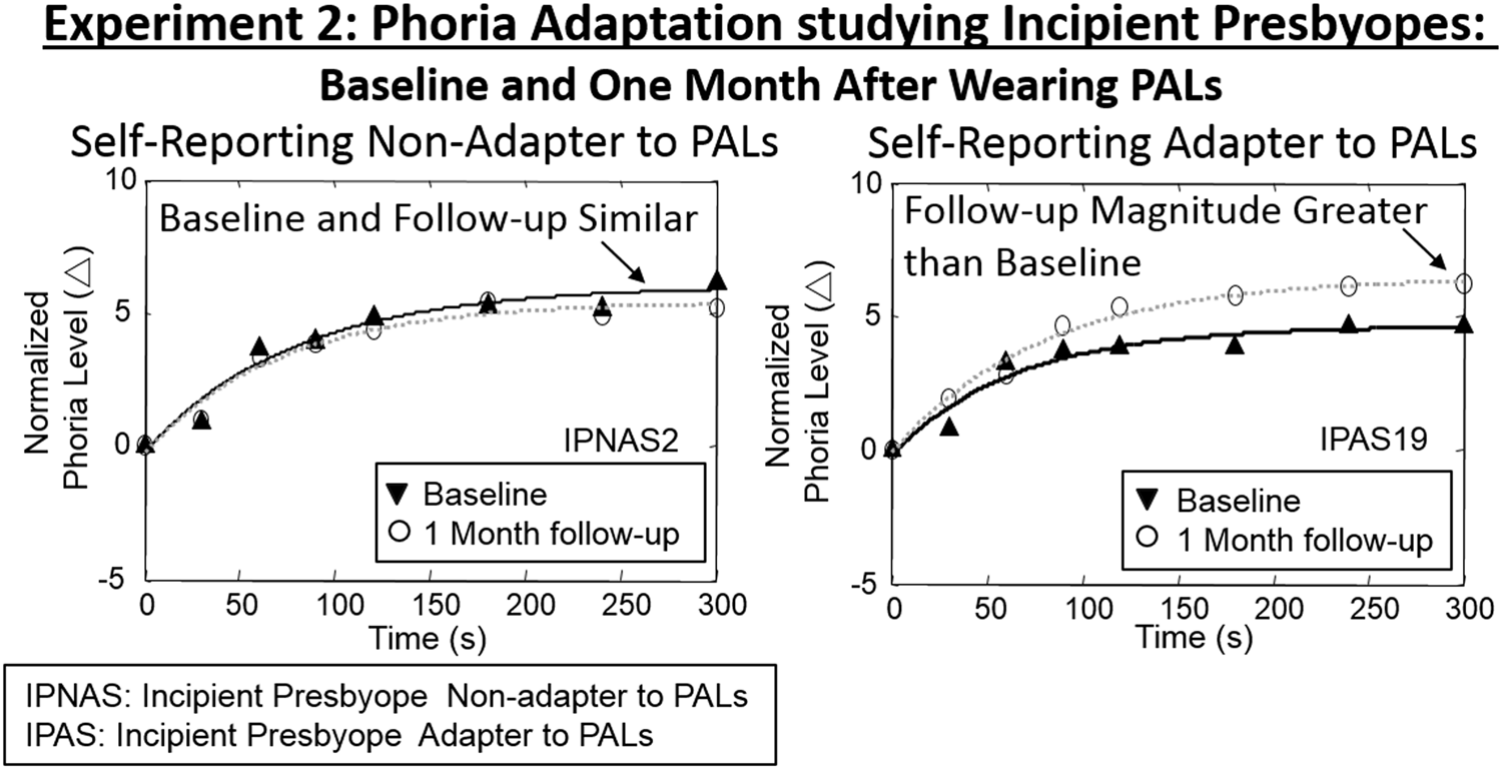
Normalized phoria measurements with exponential fit line before wearing PALs (black triangle, black line) and after one month of wearing PALs (white circle, gray line) during 5 minutes of 8.44° binocular fixation of a subject who did not adapt to PALs (IPNAS upper plots) and a subject who adapted to PALs (IPAS lower plots). Positive values mean an esophoric change in the subject’s phoria level and negative values means an exophoric change in the subject’s phoria level.

**Figure 6:**
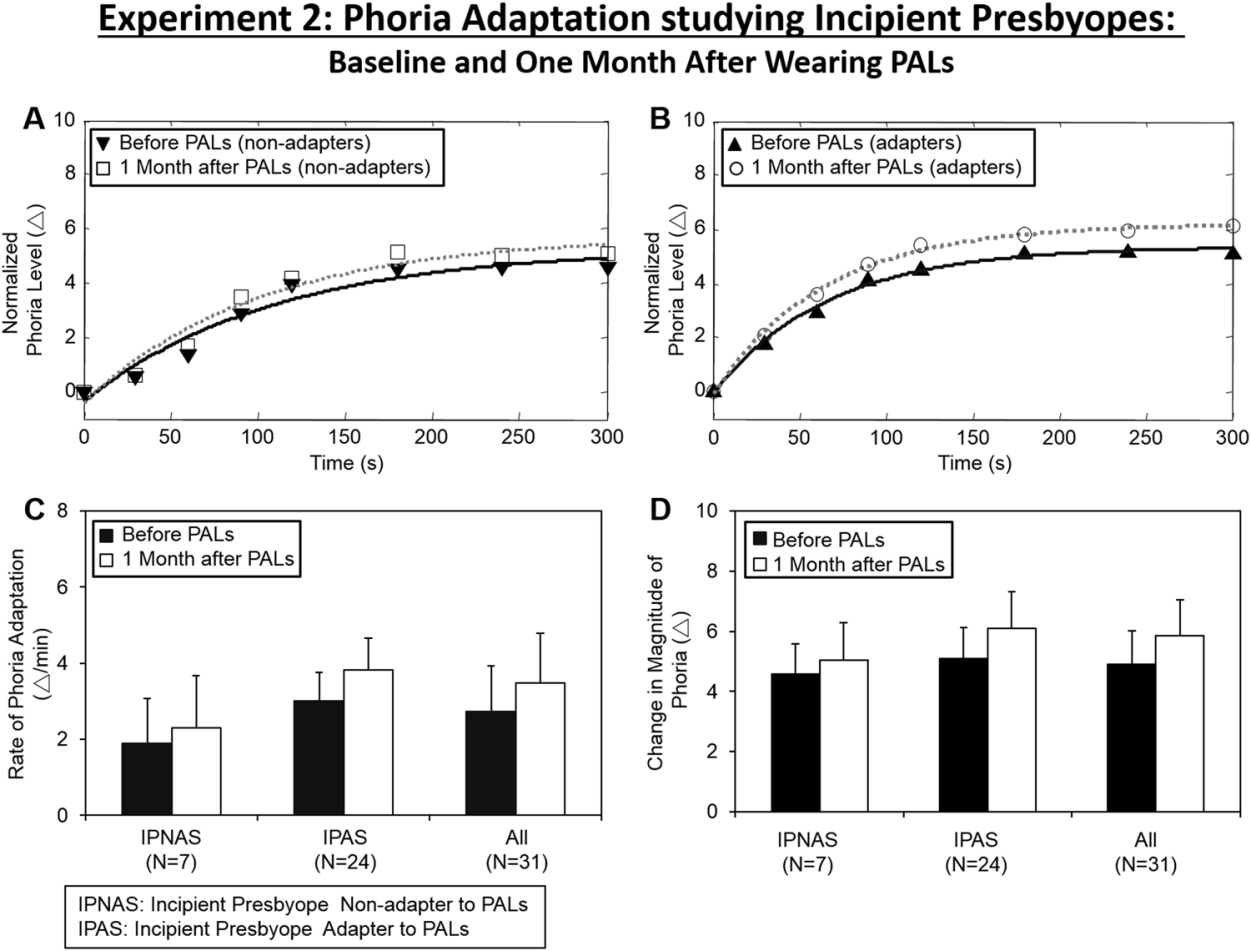
(**A**) Normalized average rate of phoria adaptation and exponential fit of IPNAS group before (upside down triangle, black line) and one month after wearing PALs (open circle, gray line). (**B**) Normalized average rate of phoria adaptation and exponential fit of IPAS group before (triangle, black line) and one month after wearing PALs (open circle, gray line). Group level average and one standard deviation of the rate of phoria adaptation (**C**) and the change in magnitude of phoria adaptation (**D**) before (black bar) and one month after wearing PALs (white bar) of IPNAS, IPAS and all subjects.

Data were normalized where all subjects’ baseline phoria measurement was removed so that the initial phoria began at zero for easier comparison between subjects. Figure 5 illustrates normalized phoria measurements with an exponential fitted curve of a series of phoria measurements in response to a 5-minute adaptation to a 40 cm (8.44°) binocular target recording during the baseline experiment and repeated after one month after wearing PALs. An average IPNAS subject is shown in Fig. 5 (left) and an average IPAS subject is in Fig. 5 (right). The average rate (upper panel) and the change in the magnitude (lower panel) of phoria adaptation measured at baseline and repeated after one month of PALs treatment are summarized in Fig. 6.

A repeated-measures ANOVA across all subjects revealed a significant increase in the magnitude [F(1,60) = 7.69, *p* < 0.01] but not the rate of phoria adaptation [F(1,30) = 2.65, *p* > 0.10] after wearing PALs for one month compared to the baseline measurement. The mean and standard deviation of the change in magnitude and the rate of phoria adaptation during the baseline measurements were 4.91 ± 0.99Δ and 2.72 ± 1.18Δ/min compared to the measurements recorded after wearing PALs for one month, which were 5.86 ± 1.24Δ and 3.47 ± 1.37Δ/min, respectively. A repeated-measures of ANOVA demonstrated a significant change in the magnitude of phoria adaptation for the PNAS group [F(1,41) = 11.76, *p* < 0.01]. However, there were no significant differences in the change in magnitude among the IPNAS group [F(1,13) = 1.94, *p* > 0.1].

Figure 6 illustrates normalized and averaged phoria measurements collected at baseline and after wearing PALs for one month for both the IPNAS (plot 6 A) and IPAS (plot 6B) groups. Group means and standard deviations for rate and change in magnitude of phoria adaptation, both before and after one month of wearing PALs, are shown in Fig. 6 plots C and D, respectively. An unpaired *t*-test showed that the rate of phoria adaptation in the IPNAS group was significantly different compared to the IPAS group for both the measurements recorded at baseline (t = 2.35, *p* = 0.026) and for those recorded one month after wearing PALs (t = 2.43, *p* = 0.021). The magnitude and rate of phoria adaptation were greater within the IPAS compared to the IPNAS group. No significant difference was observed for the change in magnitude between the two groups at baseline (t = 0.79, *p* = 0.43). However, a significant difference between groups was observed after one month of wearing PALs (t = 2.37, *p* = 0.025).

### Clinical correlates of rate and change in magnitude of phoria adaptation

**Figure 7:**
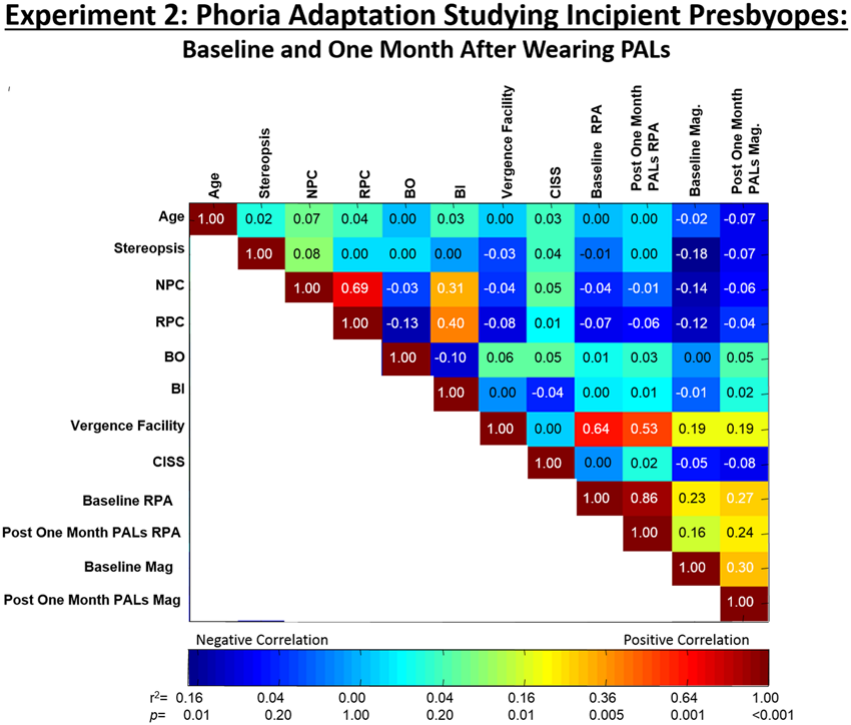
Correlation and statistical significance analysis between all parameters measured during the data collection. Pearson’s correlation coefficient values (r^2^) are displayed in each bin. The color map shown below correspond to the r^2^ and *p* values. NPC: near point of convergence, RPC: recovery point of convergence, BO: base-out vergence range, BI: base-in vergence range, CISS: convergence insufficiency symptom survey score, RPA: rate of phoria adaptation, Mag: magnitude of change in phoria, Baseline: Before wearing PALs, Post One month after wearing PALs. Positive correlation is denoted in red and negative correlation is denoted in blue. Data are from Experiment 2 studying incipient presbyopes.

The results of linear regression analyses between age, five clinical parameters, the rate and the change in magnitude of phoria from the baseline session 1 and after wearing PALs for one month for session 2 are shown in Fig. 7. Several significant correlations were observed. First, the change in the magnitude of phoria before wearing PALs were significantly correlated with stereopsis (r^2^ = 0.18, *p* = 0.03) and near point of convergence (r^2^ = 0.14, *p* = 0.03). Secondly, there was a moderate to large correlation between vergence facility and the change in magnitude of phoria (r^2^ = 0.19, *p* < 0.01), as well as the rate of phoria adaptation (r^2^ = 0.64, *p* < 0.001). Lastly, the rate and the change in the magnitude of phoria were significantly correlated (r^2^ > 0.16, *p* < 0.01).

An unpaired *t*-test demonstrated no statistical differences between groups for stereopsis, near point of convergence, recovery point of convergence, base-in vergence range, base-out vergence range, vergence facility or CISS score (T < 1.30, *p* > 0.2). Although the *t*-test did not reach significance (*p* = 0.30), IPAS subjects showed better vergence facility as compared to IPNAS subjects (IPNAS: 7.3 ± 4.0 cpm, IPAS: 11.01 ± 8.2 cpm).

**Figure 8:**
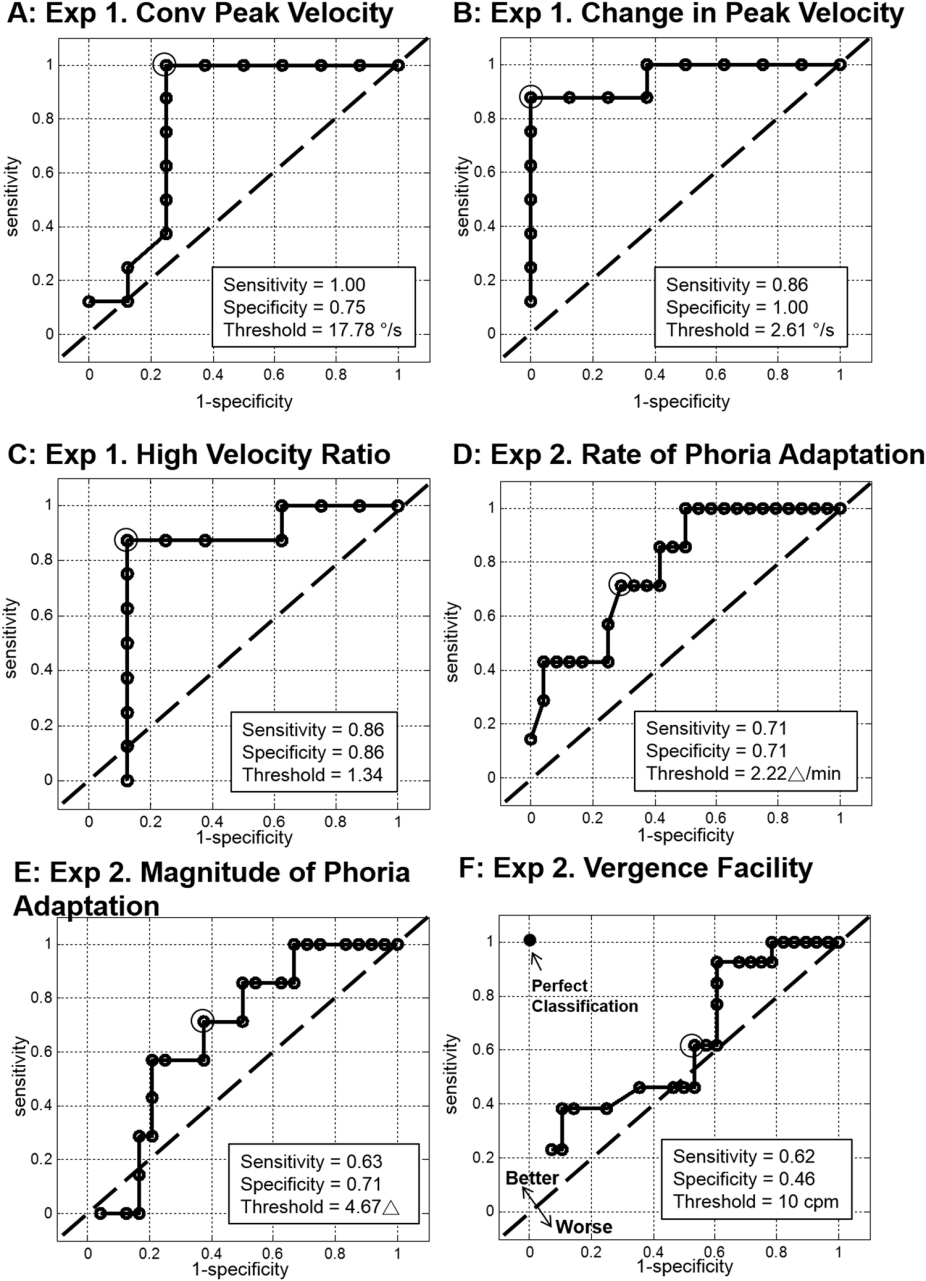
The line of no discrimination (dashed black line) and the receiver operating characteristic (ROC) curves (solid black line) with the optimal threshold (indicated as gray circle) for PALs acceptability for the following parameters: (**A**) baseline peak velocity (accuracy = 75%), (**B**) change in convergence peak velocity (accuracy = 88%), (**C**) high-velocity ratio (accuracy = 88%), (**D**) rate of phoria adaptation (accuracy = 71%), (**E**) change in phoria magnitude (accuracy = 71%), and (**F**) vergence facility (accuracy = 73%).

Figure 8 plots the receiver operating characteristics (ROC) curves of six parameters: 4° convergence peak velocity during baseline (plot 8 A), the change in peak velocity during the short-term vergence modification experiment (plot 8B), the high-velocity ratio (plot 8C), the rate (plot 8D) and the change in magnitude of phoria (plot 8E), and vergence facility (plot 8F). Numerous thresholds were analyzed and the optimal sensitivity, specificity and accuracy are tabulated in Table 3 for the optimal threshold studied.

**Table 3:** Specificity, sensitivity, accuracy and the optimal threshold for the following parameters studied.

Parameter	Specificity	Sensitivity	Accuracy	Optimal Threshold
Baseline Convergence Peak Velocity (deg/s)	75%	100%	88%	17.78 deg/s
Change in Peak velocity (deg/s)	100%	86%	94%	2.61 deg/s
High Velocity Ratio	86%	86%	88%	1.34
Rate of Phoria Adaptation (Δ/min)	71%	71%	71%	2.22 Δ/min
Change Magnitude of Phoria Adaptation (Δ)	71%	63%	64%	4.67Δ
Vergence Facility (cycle per min (cpm))	42%	62%	48%	10 cpm

## Discussion

The novelty of this study was investigating the ability to modify the disparity vergence system assessed using experiment 1 as well as the rate and magnitude of phoria adaptation assessed using experiment 2 as factors to consider when prescribing PALs to patients. Currently, eye care professionals do not have a standardized technique to determine which patients will likely adapt to PALs and which patients will have a difficult time. We consider three main parameters for patients who are prescribed PALs, namely an increased ability to change 4° disparity-convergence peak velocity during a short-term modification experiment, an increased rate of phoria adaptation, and greater vergence facility.

### Short-term modification of convergence in PALs acceptability

Previous studies have reported that the disparity-vergence system is not influenced by age^68–70^, although decreases in disparity-vergence peak velocity with age has been reported^71^. Subject-dependent variations in the ability to adapt vergence responses has also been shown in previous studies^33, 49, 72, 73^. The results of this study support that subject-dependent variations in convergence peak velocity as well as the ability to change convergence peak velocity were measurements that could be considered for PALs acceptance. For example, non-adapters to PALs demonstrated slower convergence peak velocity and the reduced ability to change convergence responses compared to subjects who adapted to PALs. It is speculated that since accommodative convergence decreases rapidly with the onset of presbyopia^26^, the adaptability of disparity-vergence may be critical in maintaining single binocular vision in a visually changing environment such as when wearing PALs.

For experiment 1, it should be noted that the patients studied had a history of wearing or attempting to wear PALs. Since we do not have baseline data from these subjects, we can only speculate that habitual wearing of PALs may have potentially altered the underlying physiology.

### Rate of phoria adaptation in PALs acceptability

Phoria adaptation modifies the phoria to reduce the load on the disparity-vergence system^24, 74–76^. Previous studies have shown that sustained fixation using plus and minus lenses stimulates an exophoric or esophoric shift in phoria adaptation, respectively^77–79^. This study observed that non-adapters to PALs had a reduced ability to adapt to a visual target in near space compared to adapters of PALs. Therefore, during the initial trial period of wearing PALs, the ability to quickly adapt to visual targets at different spaces (close to a subject compared to far away) may be an important trait in presbyopes adapting to PALs.

Previous studies have yet to examine differences in phoria adaptation before and after one month of wearing PALs within the presbyopic population. This study reports that adapters to PALs demonstrated a significant increase in the change in the magnitude of phoria adaptation compared to non-adapters to PALs. Decreases in the change of the magnitude of phoria during phoria adaptation have been suggested as one of the potential causes of asthenopia or visual complaints in those with normal binocular vision^80^ as well as patients with binocular dysfunctions such as convergence excess^81, 82^ and convergence insufficiency^81, 83–85^. Based upon these studies, the underlying visual complaints that are commonly experienced by non-adapters to PALs may be associated with a decrease in the rate of phoria adaptation and perhaps the reduced ability to change the magnitude of phoria after one month of wearing PALs.

### Vergence facility and PALs acceptability

Vergence facility has been studied in presbyopic^86^ and non-presbyopic^61^ populations. When using prisms of 8Δ base-in and 12Δ base-out prisms, only 21 out of 42 presbyopic subjects were able to perform vergence facility^86^. Significant differences in vergence facility performance were observed in visually symptomatic and non-symptomatic non-presbyopic group using prisms of 3Δ base-in and 12Δ base-out while fixating on a near target (40 cm)^87^. They report that vergence facility measurements were highly repeatable after 30 or more days (r^2^ = 0.72) and that a threshold of 15 cycles per minutes (cpm) yielded highest sensitivity (75%) and specificity (85%) for correctly identifying symptomatic and non-symptomatic subjects. Subsequent studies have suggested that the vergence facility measurement reflects dynamic properties of the vergence system since vergence facility measurements were not correlated with other static clinical vergence parameters binocular vision (for example base-out /base-in proximal fusional range to blur and break point^88, 89^). McDaniel and Fogt reported that phoria changes before and after vergence facility measurements (n = 30, 3.25Δ ± 1.58Δ) and that vergence facility may be affected by phoria adaptation^89^. The results of this study support that vergence facility was highly correlated with the rate of phoria adaptation and moderately correlated with the change in magnitude of phoria adaptation. Based upon this study’s results, vergence facility may reflect the adaptability of both vergence and phoria systems.

### Other Factors influencing PAL Acceptance

While this present study used two experiments (experiment 1: short-term vergence motor learning and experiment 2: phoria adaptation), other factors should be considered when evaluating PALs acceptance. The proper fit of the lens is critical for the acceptance of PALs. To ensure our subjects were properly fit with the PALs prescription, an Optometrist and Optician each with over 20 years of experience ensured that the PALs were properly fitted to the subjects. Other studies report that prescription, frame size, fitting parameters and free-form manufacturing to design patient specific PALs do impact objective visual performance^90^. While our results do not show that the interpupillary distance was correlated to PALs acceptance, future studies may wish to provide a more detailed studied that concentrates on a larger distribution of interpupillary distances. Prior research has also shown that different types of PALs can lead to different amount of aberrations where high-order aberrations are observed around the corridor area and the near zone in PALs based upon an optical property analysis^91^. Gambra and colleagues results support that the presence or absence of high-order aberrations caused by PALs alter the accommodative response and the fluctuations of accommodation where the presence of larger amounts of high-order aberrations produce accommodative lag^20^. Sawides and colleagues support that high order aberrations and astigmatism including the distortion produced by the astigmatism^92, 93^ may also influence the adaptation to PALs. In addition, the PALs design can introduce undesirable astigmatism within the periphery that will degrade vision and negatively impact PALs adaptation^94^.

### Study limitations

It is important to note that the magnitude of phoria adaptation showed a statistical trend in its decrease with age, which has been shown to be significant in other studies^95, 96^. The current study had a smaller age range which may explain why statistical tests did not reach significance. Statistical significance may be observed in a population with a larger age range.

Additionally, classification of non-adapters and adapters was based upon self-report. These self-reported responses involve psychological factors which may affect their validity, such that subjects may exaggerate or under-report the severity and frequency of their symptoms^97, 98^. It is unknown as to whether these biases may have contributed to this study’s dataset. For example, subjects who demonstrated a lower rate of phoria adaptation (<2.26/min) may report that they have adapted to PALs but may still experience asthenopic symptoms, indicating that they have not fully adjusted to the lenses. Even with this potential bias, this study suggests that there are significant behavioral differences in the adaptability of convergence and phoria adaptation between non-adapters and adapters to PALs.

It is potentially possible that if subjects who reported they could not adapt to PALs were to wear PALs longer perhaps then these subjects may have eventually adapted to PALs. However, it is beyond the scope of this study to determine whether subjects who self-reported that they could not adapt to PALs after 1–3 months would adapt to PALs if they were given more time to wear them.

## Conclusion

Non-adapters to PALs demonstrated slower peak velocities in convergence responses to 4° disparity-vergence stimuli, a weaker ability to modify convergence responses, a reduced rate and magnitude of phoria adaptation, and a reduced vergence facility compared to adapters. The ability to change convergence peak velocity had the greatest sensitivity and specificity compared to the other parameters. However, vergence facility has potential for use in the clinic because of its ease of administration, the amount of time required to conduct the test, and the cost of required instrumentation. These results suggest that when the accommodative system decreases in presbyopic subjects, the adaptive role of vergence and phoria systems may become critical when adapting to new visual environments such as those created when using PALs.
